# Dr. Frederick B. McKenney

**Published:** 1906-04

**Authors:** 


### OBITUARY.

---

Dr. Frederick B. McKenney, of Varysburg, N. Y., died at his home in that village, March 17, 1906, aged 33 years. Dr. McKenney graduated from the University of Buffalo, Medical Department, in the class of 1898.

